# Mad Dogs

**Published:** 1872-12

**Authors:** 


### MAD DOGS

Are found quite as frequently in winter as in summer. There seems to be good reason to believe that the disease called hydrophobia is purely a nervous affection, and can be brought on by the imagination. Many imagine that mad dogs bark and foam, and cannot drink water; hence it is not uncommon for hydrophobic persons to foam at the mouth, to imitate a dog's bark, and to go into convulsions at the sight of water, or on attempting to drink it. Mad dogs do not bark, or froth, or fall into convulsions, as natural or necessary symptoms of disease. The dog should never be killed, but got into a room, if possible, and let alone, to rest or get quiet; then introduce food and drink as unobtrusively as possible; in very many cases the dog will recover of his nervousness, thus showing that the animal was not really mad, the result being that the person bitten would be relieved of an incalculably heavy mental burden. It would be well to have the mind impressed with the real symptoms of canine madness; immediately after the dog becomes mad, —

1st. He is agitated and restless, turning himself round and round all the time; goes back and forth, stopping now and then, as if uncertain what to do; he bites and snaps and howls, sometimes dashing himself against the wall; his master's voice seems to break in upon a reverie, and wake him up to a natural condition for a few moments.

2d. He does not run about, but eats and drinks, and gnaws any textile fabric he can get hold of, — rope, rag, anything.

3d. He moves his paws against the sides of his open mouth.

4th. There is a striking change in his voice.

5th. He begins to fight with other dogs, however peaceable his disposition formerly. With these symptoms he should be shot at once, if he has bitten no one, or at least chained, for he is certainly mad.

The famous mad stone is of various sizes and shapes and colors; but it is always porous, absorbing like a sponge; hence, if pressed on a bitten part, it may absorb the virus of the dog, and thus prevent an attack of hydrophobia. It should be used when possible, even if it is but a means of calming the fears, and inspiring hopefulness.

